# Heat Treatment Induced Specified Aggregation Morphology of Metoprolol Tartrate in Poly(ε-caprolactone) Matrix and the Drug Release Variation

**DOI:** 10.3390/polym13183076

**Published:** 2021-09-13

**Authors:** Zhiyu Liu, Hangling Song, Xia Chen, Aichun Han, Rong Chen, Guiting Liu, Shaoyun Guo

**Affiliations:** The State Key Laboratory of Polymer Materials Engineering, Polymer Research Institute of Sichuan University, Sichuan Provincial Engineering Laboratory of Plastic/Rubber Complex Processing Technology, Sichuan University, Chengdu 610065, China; liuzhiyu2580@gmail.com (Z.L.); shllove@163.com (H.S.); chenxia_0204@163.com (X.C.); 18202884996@163.com (A.H.); nic7702@scu.edu.cn (S.G.)

**Keywords:** aggregation morphology, drug diffusion channel, drug release variation, hot-melt blending, isothermal heat treatment, metoprolol, poly(ε-caprolactone)

## Abstract

Hot-melt blending has been widely used in the pharmaceutical industry to produce drug delivery systems, however, realizing the controlled drug release behavior of a hot-melt blended medicament it is still a tough challenge. In this study, we developed a simple and effective heat treatment method to adjust the drug release behavior, without the addition of any release modifiers. Thin metoprolol tartrate (MPT)/poly(ε-caprolactone) (PCL) tablets were prepared through hot-melt processing, and different morphologies of MPT were obtained by altering processing temperatures and the following heat treatment. MPT particles with different particle sizes were obtained under different processing temperatures, and fibrous crystals of MPT were fabricated during the following heat treatment. Different morphological structures of MPT adjusted the drug diffusion channel when immersed in phosphate-buffered saline (PBS), and various drug release behaviors were approached. After being immersed for 24 h, 7% of the MPT was released from the blend processed at 130 °C, while more than 95% of the MPT were released after the following heat treatment of the same sample. Thus, flexible drug release behaviors were achieved using this simple and effective processing manufacture, which is demonstrated to be of profound importance for biomedical applications.

## 1. Introduction

Hot-melt blending has been widely used in the pharmaceutical industry to produce drug delivery systems, by virtue of the advantages such as solvent-free, continuous, and efficient processing [1,2,3]. Due to the easily tailored processing, different dosage forms including granules [4], pellets [5], tablets [6], and films [7] have been manufactured to meet various drug delivery requirements [8,9]. With the development of clinical medicine, a drug delivery system is often required to achieve controlled drug release in order to maintain a stable blood drug concentration, reduce the side effects of drugs, and achieve a high therapeutic effect. However, realizing the controlled drug release behavior of a hot-melt blended medicament is still a tough challenge.

Up to now, numerous attempts have been made to adjust the drug release behavior of hot-melt blended medicaments, mostly focusing on the selection of polymer matrix and the interaction between polymer matrix and loaded drug. In general, the drug release behavior is subject to three aspects: the water solubility of the loaded drug, the permeability of the drug through the polymer matrix, and the biodegradability of the polymer matrix [10]. In order to avoid burst drug release, hydrophobic biodegradable polymers with low water permeability and excellent hot working performance (such as poly(ε-caprolactone), (PCL) and polylactic acid (PLA)) are frequently utilized as matrix candidates. Furthermore, release modifiers such as poly(vinylpyrrolidone) (PVP) [11], poly(ethylene oxide) (PEO) [12], and poly(ethylene glycol) (PEG) [10] are often used to adjust the drug release behavior [13]. When immersed in a simulated fluid, these release modifiers are dissolved to form different diffusion channels for the permeation and diffusion of the loaded drug. The content and the morphology of the release modifiers significantly adjust the drug release behavior [14]. However, the addition of the additives usually sacrifices other properties of the medicament such as the biodegradability and mechanical performance of the polymer matrix, complicate the hot-melt blending processing, and may even lead to toxic and side effects. Thus, the achievement of the controlled drug release behavior of a hot-melt blended medicament without release modifiers is necessary and of profound importance for the pharmaceutical fields.

In recent years, metoprolol tartrate (MPT), as a cardioselective beta-blocker, has been widely used in the treatment of hypertension, angina pectoris, cardiac arrhythmias, myocardial infarction, and other diseases [15]. However, oral use of MPT is limited by the low systemic bioavailability and short half-life period [16]. The controlled release of MPT can improve the selectivity of the pharmacological action, stabilize the plasma concentration in vivo, lower the blood pressure, and decrease the administration frequency [17]. There is an urgent need to develop a controlled drug delivery system. Different types of PCL-based matrices have been broadly used in the pharmaceutical industry as effective drug carriers. Release modifiers such as hydrophilic components were usually added to create diffusion channels and achieve appropriate release profiles of drugs [18]. In order to avoid the side effect brought by the release modifiers, tailorable drug delivery systems were manufactured through the variation of drug morphologies, instead of the addition of any additives.

In this work, we developed a simple and effective heat treatment method to adjust the drug release behavior, without the addition of any release modifiers. In this method, by adjusting the melt blending and the following heat treatment, the aggregation and crystalline morphology of the loaded drug in the polymer matrix could be controlled, and thus the diffusion channel of the drug could be specified, and the controlled drug release behavior could finally be achieved. MPT and poly(ε-caprolactone) (PCL) were selected as model drug and polymer matrix, respectively. The morphological structures of the MPT in PCL matrix treated by various melt blending and the following heat treatments were detected, and the effect of MPT morphology on the release behavior and mechanism were investigated. This heat treatment method is effective for a series of polymer/drug systems, for which the drug needs to be melted near the polymer processing temperature, and different physical properties can be realized under different heat treatments. This method could provide a versatile and robust strategy to prepare drug delivery systems with controlled drug release behavior.

## 2. Materials and Methods

### 2.1. Materials

Poly(ε-caprolactone) (PCL, CAPA6500), with M_w_ = 50,000, was obtained from Perstorp Corp. (Warrington, UK). Metoprolol tartrate (MPT) was provided by Guangzhou Hanfang Pharmaceutical Co., Ltd. (Guangzhou, China). Phosphate-buffered saline (PBS, pH = 7.2–7.4) was obtained from Zhongshan Jinqiao Corp. (Beijing, China). All materials were used as received.

### 2.2. Melt Blending and Hot-Pressing

The PCL/MPT blends were premixed in a high-speed mixer at 900 ± 5 rpm for 5 min at room temperature. Then the mixtures were mixed in an internal mixer (RM200C, Hapro Electric Technology Co., Ltd., Harbin, China) at 50 rpm for 8 min. Two mixing temperatures, 100 and 130 °C, were selected to investigate the effect of processing temperature on the drug release behavior. The blends were vacuum-dried at 40 °C for 24 h, cut into small pieces, and then compression-molded on a compression molding machine (HP-63, Xima Weili Plastic Machine Factory, Swatow China) at 100 °C under 10 MPa for 5 min; cooling was accomplished by water through the platens at 25 °C under 10 MPa for 10 min. Thin tablets were obtained, with the thickness being 0.8 mm and the diameter being 10 mm.

The compositions of the investigated matrices are summarized in Table 1. The nomenclature for the drug carriers includes the weight content of MPT and the processing temperature. For example, MPT5-100 denotes a PCL/MPT system with the weight ratio of PCL/MPT being 95/5 and the processing temperature being 100 °C. All the samples were vacuum-dried at 37 °C before use.

### 2.3. Heat Treatment

Sample MPT5-130 with a thickness of 0.8 mm and a diameter of 10 mm was placed in an 80 °C oven and isothermally treated for 1 h. The heat-treated sample (named MPT5-130-re) was stored at 25 °C before use.

### 2.4. Thermal Gravimetry Analysis (TGA)

The thermal stability of the PCL/MPT blends was examined using a TGA Q500 analyzer (TA Instruments, New Castle, DE, USA). The measurements were taken in nitrogen flow at a heating rate of 10 °C/min with temperature range from 30 to 600 °C.

### 2.5. Fourier Transform Infrared Spectroscopy (FTIR)

The structure variation of the PCL/MPT blends during the hot-melt processing was evaluated using an infrared spectrometer (IS10, Thermo Nicolet, Waltham, MA, USA). The samples were cut into thin slices by a rotary microtome (YD-2508B, Jinhua Yidi Medical Equipment Factory, Jinhua, China), with a thickness of 10 μm. Background and the samples were sequentially measured from 4000 to 400 cm^−1^ with 32 scans for each sample, at a resolution of 2 cm^−1^.

### 2.6. Scanning Electron Microscopy (SEM)

The morphology of the PCL/MPT blends was observed in a JSM-5900LV scanning electron microscope (JEOL Ltd., Tokyo, Japan) under an accelerating voltage of 20 kV. The samples were first immersed in liquid nitrogen and cryo-fractured along the thickness direction. The cross-section was washed 3 times with deionized water and vacuum-dried at 37 °C until the weight was constant. A thin layer of Pd-Au alloy was coated on the cross-section of the specimen prior to observation to prevent charging on the surface. Pore size distribution was evaluated using the image analysis program ImageJ. SEM images of the PCL/MPT blends were used for the image analysis.

### 2.7. Differential Scanning Calorimetry (DSC)

The crystalline variation of the samples during the hot-melt processing was assessed using a Q20 DSC instrument (TA Instrument Co., New Castle, DE, USA) under a nitrogen atmosphere. The samples (6–8 mg) were heated from −40 to 130 °C at a rate of 10 °C/min and maintained for 3 min. Then the samples were cooled down to 0 °C at a cooling rate of 10 °C/min and maintained for 3 min. After that, the samples were reheated to 140 °C at a rate of 10 °C/min. The degree of crystallinity (X_c_, %) for PCL and MPT in each sample was calculated as follows [19]:

X_c_ (%) = ΔH_m_/(ΔH^0^_m_ × W_x_) × 100%, (1)

where ΔH_m_ is the enthalpy of PCL or MPT obtained from the second heating process and ΔH^0^_m_ is the theoretical enthalpy of the completely crystalline PCL (135.5 J/g [20]) or MPT (106.9 J/g [21]). W_x_ is the weight fraction of PCL or MPT in the blend samples.

### 2.8. Wide-Angle X-ray Diffractometry (WAXD)

The crystalline structures of the samples were measured by a Philips X’pert Pro MPD wide-angle X-ray diffractometry (Philips, Eindhoven, The Netherlands), using Cu Kα X-rays at 40 kV and 40 mA. The 2θ values ranged from 5 to 50°, and the scanning speed was 2°/min.

### 2.9. Optical Microscope (OM)

Thin slices of the PCL/MPT blends with a thickness of 15 μm were obtained by a rotary microtome (YD-2508B, Jinhua Yidi Medical Equipment Factory, Jinhua, China) along the thickness direction of the samples. The drug morphologies of the blends were observed using an Olympus BX51 optical microscope (Japan) equipped with a camera.

In order to study the crystallization growth of MPT in the PCL matrices, a hot-stage OM was utilized. The specimen was placed between a microscope glass slide and a coverslip and heated on a hot stage (HCS302, Instec Inc., Boulder, CO, USA). The samples were heated from 25 to 80 °C at a rate of 30 °C/min and kept for 30 min for the growth of the MPT crystals.

### 2.10. In Vitro Drug Release

In vitro drug release from the drug-loaded tablets (thickness: 0.8 mm, diameter: 10 mm) was measured for up to 35 days. The tablets were incubated in 10 mL PBS at 37 °C in an incubator (HZQ-X100, Haocheng Co., Changzhou, China) stirred at 100 rpm. At each time period selected for measurement, the solution was totally exchanged with fresh PBS. All experiments were performed in triplicate. The concentrations of MPT were measured through an ultraviolet–visible spectrophotometer (UV-1750, Shimadzu Corp., Tokyo, Japan), with the wavelength set at 222 nm [1]. A standard calibration curve for MPT was prepared at concentrations ranging from 0.5 to 15 μg/mL. This curve exhibited linear behavior over the full range of concentration. If the MPT concentration of the collected solution exceeded 15 μg/mL, the solution was first diluted (to the range of 0.5–15 μg/mL), measured, and then multiplied by the dilution ratio.

### 2.11. Weight Loss

The weight loss of drug-loaded tablets during immersion was measured for up to 35 days using 40 mg of tablets incubated in 10 mL PBS at 37 °C in an incubator stirred at 100 rpm. The tablets were withdrawn at selected timepoints and vacuum-dried at 37 °C until the weight was constant. The weight of the tablets after immersion was measured, and the weight loss was calculated as follows:

Weight loss (%) = (W_0_ − W_1_)/W_1_ × 100%, (2)

where W_0_ is the initial weight of the sample and W_1_ is the final weight of the dried sample after immersion.

## 3. Results and Discussion

### 3.1. Solubility Parameter Calculation

The solubility parameter (δ) has been used to predict the miscibility of drugs and excipients. It is generally accepted that compounds with a Δδ < 7.0 MPa^1/2^ are likely to be miscible. When the Δδ > 10 MPa^1/2^_,_ the compounds are likely to be immiscible. The Hansen solubility parameters of the compounds were calculated from the chemical structure using the approaches of Hoftyzer and van Krevelen [22,23,24,25]. The cohesive energy of the compounds can be divided into three types of forces: dispersion force, couple force, and hydrogen bond force. Thus, the solubility parameters are composed by the solubility parameter components of dispersion force, hydrogen bonding, and polar interactions [26,27,28,29]:

δt = (δd^2^ + δp^2^ + δh^2^)^1/2^, (3)

where δ_t_ is the total solubility parameter calculated from the various components (dispersion force δ_d_, hydrogen bonding δ_h_, and polar interactions δ_p_). δ_d_, δ_p_, and δ_h_ can be calculated as follows:

δ_d_ = ΣF_di_/V, (4)

δ_p_ = (ΣF_pi_^2^)^1/2^/V, (5)

δ_h_ = (ΣE_hi_)^1/2^/V, (6)

where F_di_ is the group dispersion component giving δ_d_, F_pi_^2^ is the group polar component, E_hi_ is the hydrogen bonding component, and V is the molar volume from Hildebrand analysis.

The solubility parameters of MPT and PCL were calculated by the group-contribution method [19,30], and the results are shown in Table 2 and Table 3, respectively.

From the results summarized in Table 2 and Table 3, the Δδ between PCL and MPT is 4.25 MPa^1/2^, less than 7 MPa^1/2^, indicating that PCL and MPT are likely to be miscible.

### 3.2. Thermal Stability and Chemical Variation

**Figure 1:**
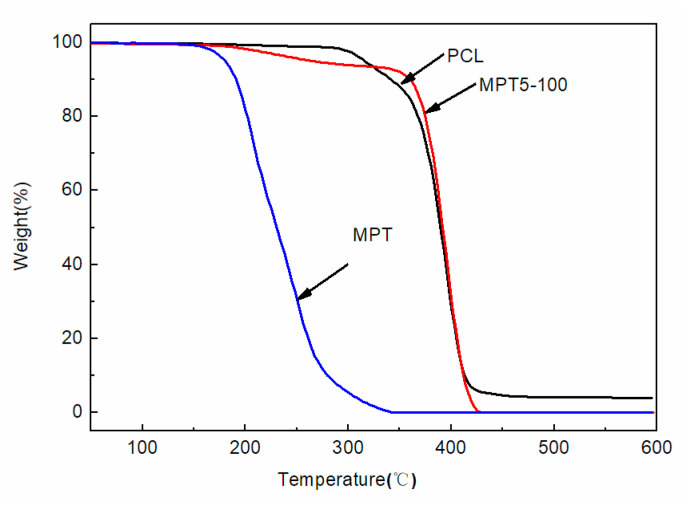
TGA curves of PCL, MPT, and PCL/MPT.

Figure 1 shows the TG curves of PCL, MPT, and the PCL/MPT blend at a heating rate of 10 °C/min under a nitrogen atmosphere. All the samples exhibited slight weight loss (less than 1%) below 160 °C, exhibiting appropriate thermal stability during the hot-melt processing (100 and 130 °C). At temperatures above 160 °C, a continuous weight loss occurred for pure MPT, indicating the degradation of MPT. The TG curve of the PCL/MPT blend has two distinct stages. The initial weight loss of about 5% in the range of 190–350 °C was attributed to the degradation of MPT. After that, a continuous weight loss occurred, possibly due to the cis-elimination reaction and the specific chain end scission of PCL [31].

**Figure 2:**
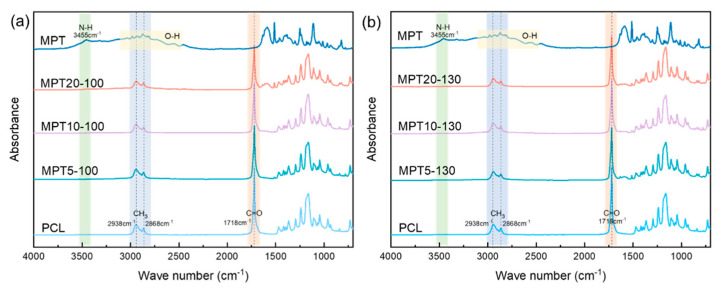
FTIR spectra of PCL/MPT blends with different processing temperatures: (**a**) 100 °C and (**b**) 130 °C.

The FTIR spectra of PCL, MPT, and the PCL/MPT blends are shown in Figure 2, with different weight ratios of PCL/MPT and processing temperatures. The bands at 2938 and 2868 cm^−1^ from pure PCL correspond to the symmetric and asymmetric stretching vibrations of -CH_3_, and the peak at 1718 cm^−1^ corresponds to the asymmetric stretching vibrations of C=O, which are in accordance with the data reported earlier [16]. The absorption band at 3455 cm^−1^ and the small broad band at 1633 cm^−1^ are related to the vibration of N-H bonding of MPT, while the broad peak ranging from 3300 to 2500 cm^−1^ is formed by the stretching vibration of O-H [32]. The FTIR spectrum of MPT is in accordance with the results reported elsewhere [33]. Compared with the FTIR curves of pure PCL and MPT, no new absorption peak appeared for PCL/MPT blends processed at different temperatures (100 and 130 °C), indicating that no chemical variations occurred during the hot-melt processing.

### 3.3. The Morphology Variation of MPT

#### 3.3.1. The Influence of the Processing Temperature

**Figure 3:**
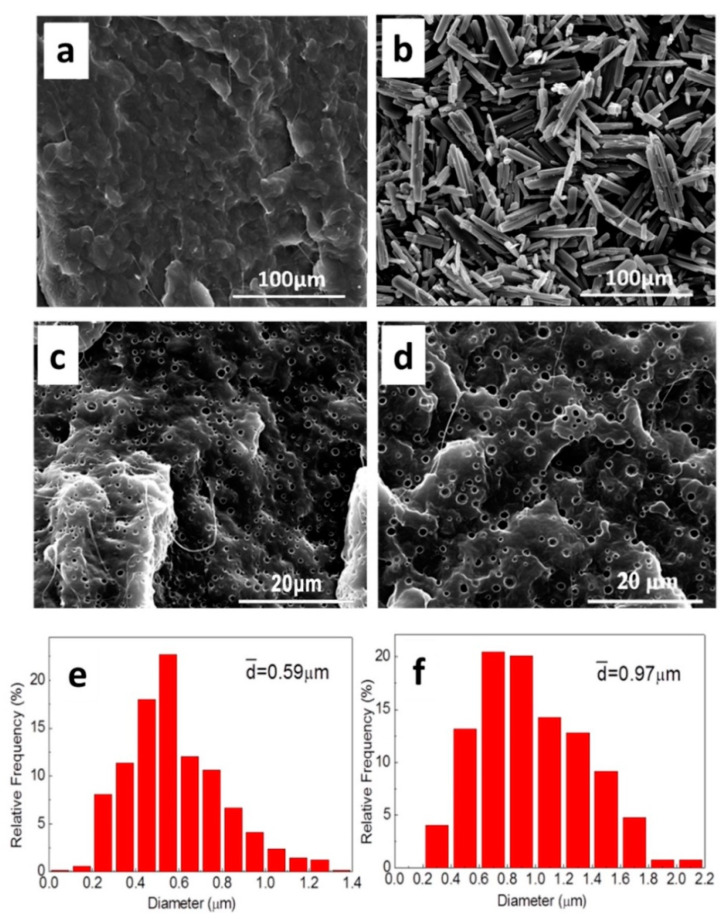
SEM images of (**a**) PCL, (**b**) MPT, and PCL/MPT after immersed in PBS for 72 h: (**c**) MPT5-100 and (**d**) MPT5-130; MPT pore size distribution: (**e**) MPT5-100 and (**f**) MPT5-130.

**Figure A1:**
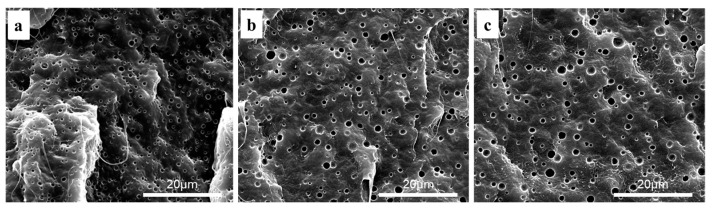
PCL/MPT after being immersed in PBS: (**a**) MPT5-100, (**b**) MPT10-100, (**c**) MPT20-100.

The SEM micrographs of PCL, MPT, and the PCL/MPT blends are shown in Figure 3, with different processing temperatures (100 and 130 °C). For PCL/MPT blends, MPT5-100 and MPT5-130 with the least content of MPT were taken as examples to discuss. Compared with the uniform and dense structure of pure PCL (Figure 3a), PCL/MPT blends (Figure 3c,d) exhibited porous structures, which is due to the dissolution of MPT during the immersion in PBS. Raw MPT particles exhibited columnar crystals (Figure 3b), while spherical structure of MPT was observed in the PCL/MPT blends (Figure 3c,d). The different morphologies of MPT suggested that even when blended at a temperature (100 °C) below the melting point of MPT (125.5 °C) [34], the strong shearing force during the processing caused the pulverization and dispersion of the MPT particles. As a result, pores with an average diameter of 0.59 μm were observed for sample MPT5-100 (Figure 3c). When the processing temperature (130 °C) is higher than the Tm of MPT (125.5 °C), the average size of MPT increased to 0.97 μm due to the particles of the melted MPT having a strong tendency to aggregate with each other to decrease the surface energy. Almeida et al. [35] had also reported that various MPT particle sizes were obtained under different processing parameters. When the MPT content became higher, MPT aggregated to form a larger dispersed phase, resulting in larger pore size (Figure A1).

The melting behaviors of PCL, MPT, and the PCL/MPT blends are shown in Figure A1. The melting temperature (T_m_), degree of crystallinity (X_c_), and heat of fusion (ΔH_m_) of the components are summarized in Table A1. The pure PCL exhibited a melting temperature of 63.6 °C, which is in accordance with the results reported elsewhere [36]. The DSC thermogram of MPT showed a clear melting endotherm at 125.5 °C, indicating its crystalline nature [35]. The Tm and X_c_ of PCL in the PCL/MPT blends were almost unchanged after being processed at different temperatures (100 and 130 °C), indicating that MPT did not affect the crystallization of PCL.

When processed at 100 °C, the Tm of MPT was slightly decreased (Figure A1a and Table A1). That is, despite the Tm of MPT (125.5 °C) being higher than the processing temperature (100 °C), an incomplete crystallization of MPT was observed. The drug–polymer interactions were evaluated to understand the blending behavior of MPT and PCL.

As we discussed above, the solubility parameter of MPT is 23.82 MPa^1/2^, and that of PCL is 19.57 MPa^1/2^. Due to the difference in the solubility parameters (Δδ = 4.25 MPa^1/2^), a partial miscibility between PCL and MPT is expected. As a result, a small amount of MPT was dissolved in the PCL matrix and did not recrystallize upon cooling, leading to a lower X_c_ of MPT (71.1% for sample MPT5-100) than that of pure MPT (99.6%). When the MPT content became higher (sample MPT20-100), MPT aggregated to form a larger dispersed phase, resulting in the enhancement of the MPT crystallization (the X_c_ of MPT is 82.3% for sample MPT20-100).

**Figure A2:**
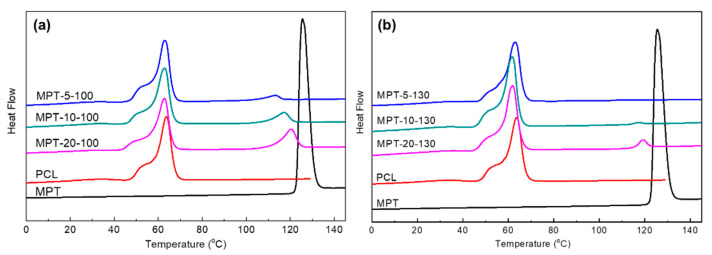
DSC heating curves of PCL/MPT blends with different processing temperatures: (**a**) 100 °C and (**b**) 130 °C.

When the processing temperature (130 °C) was higher than the T_m_ of MPT, the drug was completely melted during the processing. Due to the partial miscibility between PCL and MPT, the solubilization of MPT in the PCL matrix is more detectable. Thus, the crystallization of MPT was negligible at a low content of MPT (5%) (Figure A2 and Table A1). At higher MPT contents, the aggregation of MPT promoted the crystallization of MPT, resulting in higher X_c_ of MPT.

**Figure 4:**
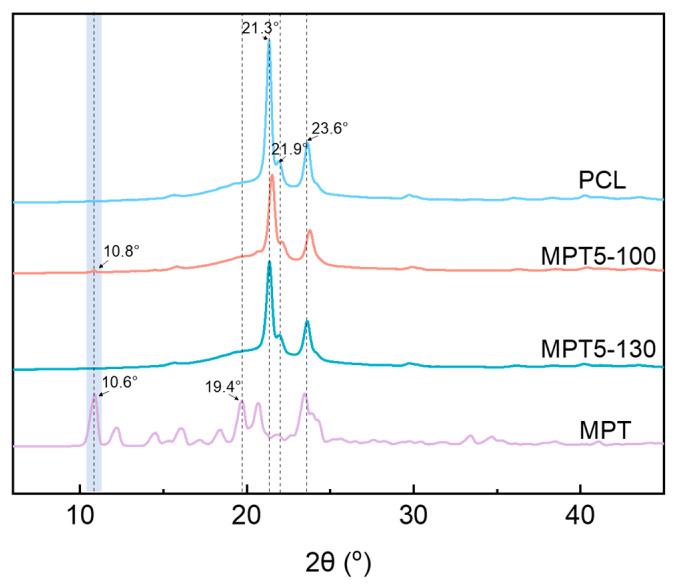
WAXD patterns for PCL/MPT-95/5 at different processing temperatures.

The WAXD diagrams of PCL, MPT, and the PCL/MPT blends are shown in Figure 4, with different processing temperatures (100 and 130 °C). The crystalline region of the PCL matrix is defined by three peaks between 20 and 25° 2θ with basal spacings of 0.414, 0.374, and 0.325 nm, which can be assigned as the 110, 111, and 200 crystal planes, respectively [37]. MPT had distinct crystalline peaks at 2θ of 10.6, 19.4, and 23.1° and a series of smaller peaks at 15.8, 20.4, and 24.0°. The results are in accordance with the results reported elsewhere [38].

**Figure A3:**
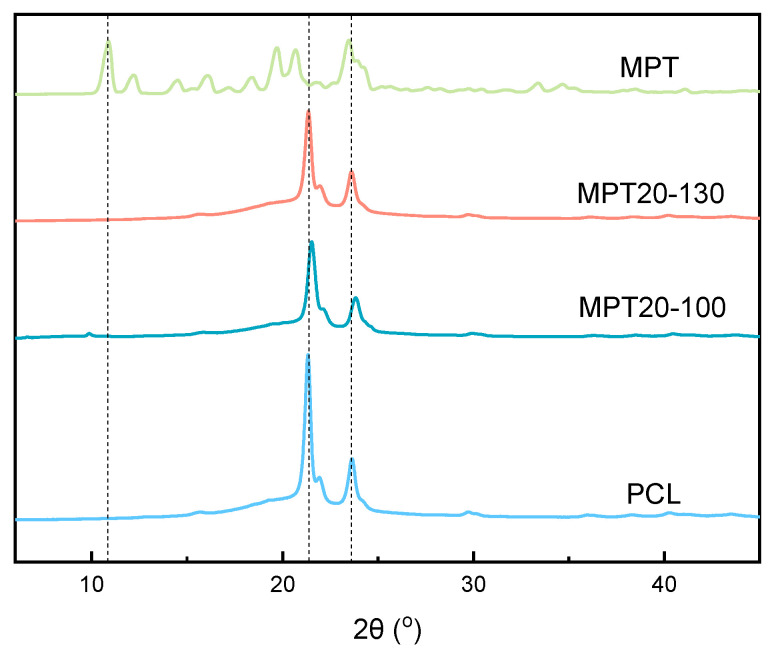
WAXD patterns for PCL/MPT-80/20 at different processing temperatures.

The WAXD pattern of the sample MPT5-100 exhibited identical peaks of PCL, indicating that the addition of 5 wt% MPT does not affect the crystallization of PCL. A slight peak at 10.8° was observed, corresponding to the crystalline conformation of MPT. When processed at 130 °C (sample MPT5-130), the weak diffraction peak of MPT at 10.8° disappeared. That is, MPT was mostly amorphous, which is in accordance with the DSC results. The same results were obtained from the WAXD patterns of samples with higher MPT contents (Figure A3).

#### 3.3.2. The Influence of the Heat Treatment

**Figure A4:**
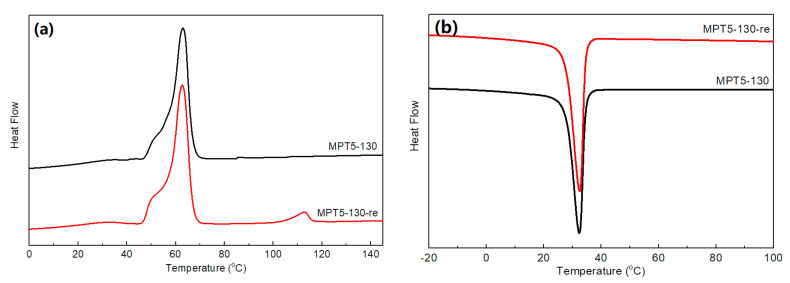
DSC curves of MPT5-130 blends for different heat treatments: (**a**) heating curves; (**b**) cooling curves.

To the best of our knowledge, heat treatment can erase the thermal history of the materials and improve their properties [39,40]. In the pharmaceutical field, heat treatment has been employed to design the drug release behaviors by morphological reconstruction of the matrices [41,42,43,44]. However, the effect of heat treatment on the drug morphology and release behavior has been rarely reported. In order to obtain different crystalline morphology of MPT, sample MPT5-130 was placed in an 80 °C oven and isothermally treated for 1 h. The heat-treated sample was named MPT5-130-re. The DSC thermograms of sample MPT5-130-re showed a visible melting endotherm of MPT at 113.0 °C (Figure A4a), indicating the recrystallization of MPT during the heat treatment process. The Tm of PCL remained the same, for the reason that the heat treatment did not affect the crystallization behavior of PCL (Figure A4).

**Figure 5:**
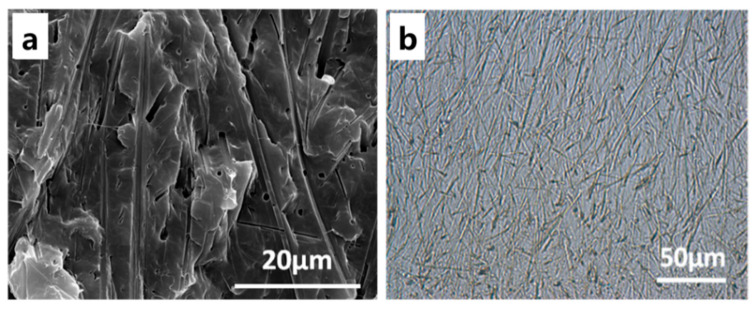
(**a**) SEM image of MPT5-130-re after being immersed in PBS for 72 h; (**b**) OM photo at 80 °C.

**Figure 6:**
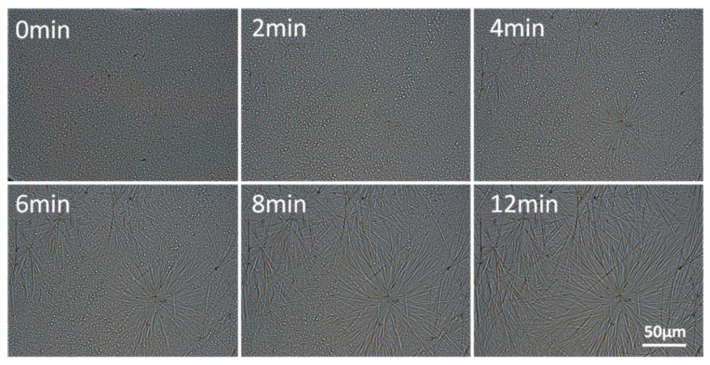
Hot-stage OM photographs of MPT5-130 isothermal at 80 °C.

OM and SEM (Figure 5) were used to evaluate the morphological variation of MPT. After isothermal treatment at 80 °C for 30 min, radial fibrous crystalline of MPT was observed in sample MPT5-130-re, indicating the recrystallization of MPT. The hot-stage OM was utilized (Figure 6) to investigate the growth behaviors of the MPT crystals during the heat treatment. At first, the MPT was dispersed as discrete particles in the PCL matrix, which was in accordance with the SEM images of MPT5-130 mentioned above (Figure 3d). After isothermal treatment for 4 min, radial-fibrous crystals of MPT appeared, exhibiting the recrystallization of MPT. Then, the fibrous crystals overlapped with each other, and the crystallization was completed.

### 3.4. In Vitro Drug Release Behavior

**Figure 7:**
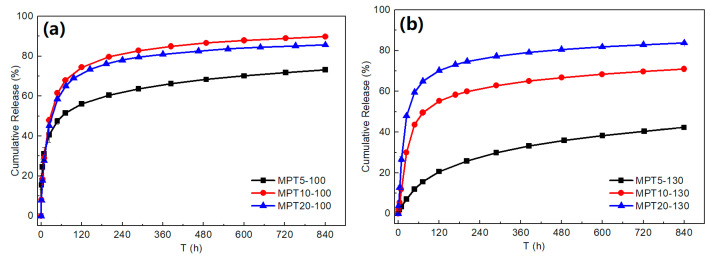
Cumulative release of MPT from the PCL/MPT blends: (**a**) 100 °C and (**b**) 130 °C.

The effect of drug loading and processing temperature on the MPT release is shown in Figure 7. About 40% of the MPT was released from sample MPT5-100 after 24 h immersion, while 48% of the MPT was released at higher drug loading (10% and 20%) (Figure 7a). When the PCL/MPT blends were fabricated at 130 °C, only 7% of the MPT was released after 24 h immersion (sample MPT5-130), and 20% of the MPT was released after 100 h. That is, the MPT release was significantly suppressed by virtue of the higher processing temperature. When the drug loading increased from 5 to 20%, the release amount of MPT increased from 7 to 48% after being immersed for 24 h (Figure 7b). Thus, both the hot-melt process and the drug content affect the MPT release behavior. Noticeably, more than 95% of the MPT was released from MPT5-130-re after 4 h immersion, exhibiting a significant burst release.

It is necessary to understand the effects of the drug morphology on the drug release mechanism and release kinetics from the PCL/MPT blends. Due to the hydrophobicity of the PCL matrix and the high dissolution of MPT in PBS [45], it is reasonable that different morphological structures of MPT caused different drug release behavior. Since MPT is easily dissolved in water [45], the outer MPT was dissolved rapidly during the immersion, leaving a large number of release channels for the diffusion of the inner drug, then resulting in the subsequent release of inner MPT. For a certain drug loading, a higher processing temperature (130 °C) caused a larger domain size of MPT (0.97 μm) (Figure 3). Agglomerate MPT decreased the overlapping of the release channels, and thus the release behavior was restricted. Likewise, larger MPT contents led to more diffusion channels for the permeation of inner MPT, and thus the drug release was significantly suppressed with the decrease of MPT content (Figure 7). After the following heat treatment, the fibrous MPT crystal was overlapped, and the interconnected release channels were formed during the immersion, promoting the MPT release.

After 35 days of immersion, pure PCL has a weight loss of 1.6 ± 0.5% [44] (Table A2), indicating that PCL did not degrade during the immersion. The PCL/MPT blends have a slight weight loss compared to pure PCL, which was mainly due to the dissolution of MPT. It was reasonable that the release mechanism of MPT was a combination of dissolution and diffusion releases. The dissolution release of MPT is attributed to the morphological structure of outer MPT, and the diffusion of inner MPT out of the PCL matrices is the diffusion release mechanism, the pores of which were caused by the dissolution of the outer MPT.

**Figure 8:**
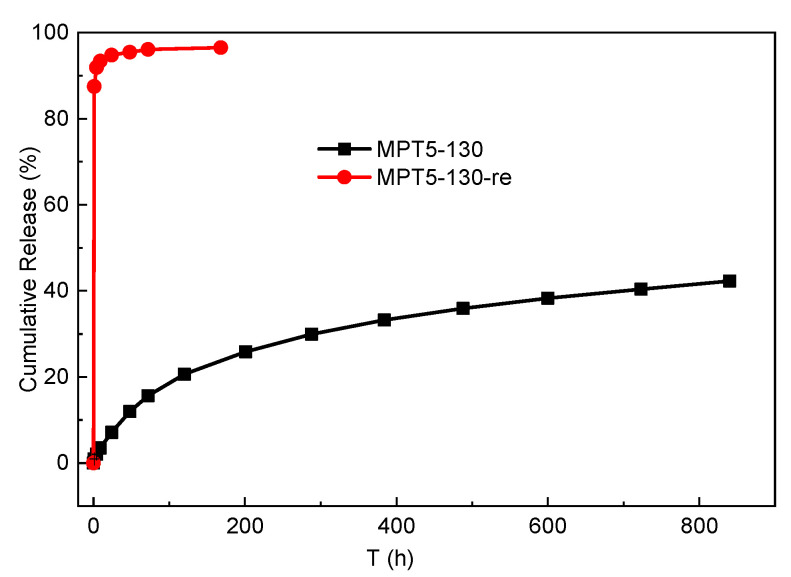
Cumulative release of MPT from the MPT5-130 after heat treatment.

For sample MPT5-130, due to the larger domain size and aggregation of MPT, the diffusion channel caused by the dissolution of MPT was unconnected, and then the initial burst release of MPT was suppressed. During the whole immersion process, the MPT release rate was constant (Figure 8).

Various release behaviors of MPT can be obtained, which can fulfill the diversified requirements in the medical therapy fields. In addition, this method offers a facile and efficient approach to construct a variety of tablets for controlled drug delivery. As a result, the release of MPT from the PCL matrix follows diffusion release kinetics during the in vitro experiment. We can control the release rate of MPT and obtain various drug release behaviors via hot-melt blending and postprocessing conditions.

### 3.5. In Vitro Drug Release Kinetics

Higuchi diffusional model was used to investigate the drug release kinetics [46]:

Q = ((D_ε_/τ) × (2C_t_ − εC_0_) × C_0_t)^1/2^ = k_H_ × t^1/2^ (7)

where Q is the amount of drug released after time t per unit surface, D is the diffusivity of the drug molecule in the permeating solution, ε is the porosity of the matrix, τ is the tortuosity factor of the capillary or pore system, C_t_ is the initial concentration of the drug in the tablet, C_0_ is the drug solubility in the pellet matrix, and k_H_ is the Higuchi dissolution constant for molecules in the liquid.

**Figure 9:**
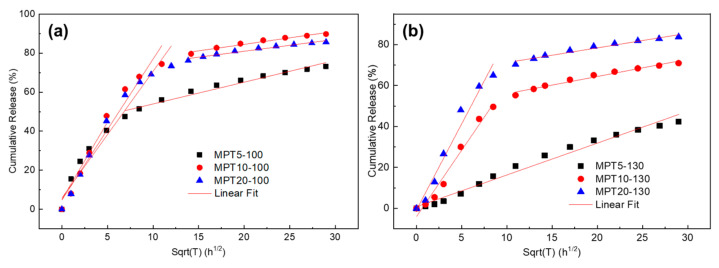
The release kinetics of PCL/MPT-100 (**a**) and PCL/MPT-130 (**b**) blends.

The release kinetics of MPT is shown in Figure 9. All the data were fitted well by the equation (Table A3). Zhang et al. discussed the mechanism and kinetics of drug release from PCL-based matrices with some release modifiers [18]. It was reported that PCL/hydrophilic drug delivery systems exhibited a two-stage release: a fast release caused by the fast dissolution of the hydrophilic components, followed by the second stage of much slower release caused by the porous structure of the PCL matrix. Parameters such as the first-order kinetic coefficient (k_1_) and the Higuchi coefficient (k_2_) can help to unravel the contribution of these two release mechanisms during the drug release. In this study, two stages of drug release were also observed, without any release modifiers. That is, water-soluble MPT at the outer space of the matrix dissolved quickly at the beginning of the immersion, leaving a large number of release channels for the diffusion of the inner drug. For sample MPT5-130, due to the higher processing temperature (130 °C) and lower MPT content (5%), fewer diffusion channels were obtained, the initial burst release of MPT was suppressed, and the MPT release rate was nearly constant during the whole immersion process.

## 4. Conclusions

In this study, thin MPT/PCL tablets with different release behaviors were obtained through hot-melt blending and heat treatment processes. Different domain sizes of loaded drug MPT were achieved via different processing temperatures. A larger domain size of MPT (processed at 130 °C) was observed compared to that of the blends processed at lower temperature (100 °C), which caused the diffusion channel to be unconnected and then suppressed the MPT release rate. In addition, the heat treatment after hot-melt blending can noticeably change the morphological structure of MPT. The recrystallization of MPT during the heat treatment generated radial-fibrous crystals, the interconnection of which formulated continuous diffusion paths to enhance the MPT release rate. More than 95% of MPT was released from sample MPT5-130 after 24 h immersion, while only 7% of MPT was release from sample MPT5-130-re after 24 h immersion. By using hot-melt blending and then heat treatment, various morphological structures of MPT and different MPT release behaviors were obtained, without adding any other additives which may not biocompatible. A continuous and environmentally friendly method was developed to formulate a series of morphological structures to fulfill various complicated medical therapies.

## Figures and Tables

**Table 1:** The formulation of PCL/MPT blends.

Specimen	PCL, wt%	MPT, wt%	Processing Temperature, °C
MPT5-100	95	5	100
MPT10-100	90	10	100
MPT20-100	80	20	100
MPT5-130	95	5	130
MPT10-130	90	10	130
MPT20-130	80	20	130

**Table 2:** Calculation of solubility parameter for MPT.

Group	F_di_	F_pi_^2^	E_hi_	V/cm^3^·mol^−1^	δ/MPa^1/2^
(2) di-substituted benzene ring	1270 × 2	110 × 2	0	52.4 × 2	23.82
(6) CH_3_	420 × 6	0	0	33.5 × 6	
(8) CH_2_	270 × 8	0	0	16.1 × 8	
(6) CH	80 × 6	0	0	−1.0 × 6	
(4) -O-	100 × 4	400 × 4	3000 × 4	3.8 × 4	
(2) NH	160 × 2	210 × 2	3100 × 2	4.5 × 2	
(4) OH	1270 × 2	110 × 2	0	10.0 × 4	
(2) COOH	530 × 2	420 × 2	10,000 × 2	28.5 × 2	
Σ	10,320	5080	118,200	549.8	

**Table 3:** Calculation of solubility parameter for PCL.

Group	F_di_	F_pi_^2^	E_hi_	V/cm^3^·mol^−1^	δ/MPa^1/2^
(5) CH_2_	270 × 5	0	0	16.1 × 5	19.57
(1) COO	390	490	7000	18.0	
Σ	1740	490	7000	98.5	

## Appendix A

**Table A1:** Thermal properties of PCL/MPT blends with different processing temperatures.

Specimen	PCL			MPT		
	T_m_, °C	ΔH_m_, (J/g)	X_c_, %	T_m_, °C	ΔH_m_, (J/g)	X_c_, %
MPT	/	/	/	125.5	106.5	99.6
PCL	63.7	63.6	47.0	/	/	/
MPT5-100	62.9	62.7	48.7	113.0	3.8	71.1
MPT10-100	62.8	57.4	47.1	117.0	8.2	76.7
MPT20-100	62.8	54.0	49.9	120.3	17.6	82.3
MPT5-130	63.0	58.8	45.7	/	/	/
MPT10-130	61.2	56.5	46.3	116.3	0.5	4.7
MPT20-130	60.4	53.7	49.5	119.3	4.8	22.5

**Table A2:** Weight loss of PCL/MPT blends after being immersed for 35 days.

Specimen	Weight before Immersion, mg	Weight after Immersion, mg	Weight Loss, %
PCL	67.5 ± 0.4	66.4 ± 0.7	1.6 ± 0.5
MPT5-100	73.0 ± 8.4	69.6 ± 7.5	4.6 ± 0.7
MPT10-100	72.8 ± 5.0	65.6 ± 4.6	9.9 ± 0.1
MPT20-100	69.0 ± 1.7	56.9 ± 1.2	17.5 ± 0.5
MPT5-130	70.8 ± 7.3	68.5 ± 7.2	3.3 ± 0.2
MPT10-130	67.6 ± 3.0	61.8 ± 3.0	8.5 ± 0.3
MPT20-130	65.5 ± 0.8	53.8 ± 0.6	17.8 ± 0.4
MPT5-130-re	60.7 ± 3.1	57.0 ± 3.4	6.0 ± 1.0

**Table A3:** Higuchi dissolution constants, k_H_, and correlation coefficients, R_2_, of PCL/MPT blends.

Specimen	First Stage		Second Stage	
	k_H_, h^1/2^	R_2_	k_H_, h^1/2^	R_2_
MPT5-100	7.93	0.94	1.12	0.96
MPT10-100	7.22	0.95	0.67	0.96
MPT20-100	6.49	0.95	0.61	0.96
MPT5-130	1.56	0.98	/	/
MPT10-130	6.48	0.97	0.84	0.97
MPT20-130	8.41	0.97	0.72	0.96
